# Vaccine coverage and adherence to EPI schedules in eight resource poor settings in the MAL-ED cohort study

**DOI:** 10.1016/j.vaccine.2016.11.075

**Published:** 2017-01-11

**Authors:** Christel Hoest, Jessica C. Seidman, Gwenyth Lee, James A Platts-Mills, Asad Ali, Maribel Paredes Olortegui, Pascal Bessong, Ram Chandyo, Sudhir Babji, Venkata Raghava Mohan, Dinesh Mondal, Mustafa Mahfuz, Estomih R Mduma, Emanuel Nyathi, Claudia Abreu, Mark A. Miller, William Pan, Carl J. Mason, Stacey L. Knobler, the MAL-ED Network Investigators

**Affiliations:** a Division of International Epidemiology and Population Studies of Fogarty International Center, National Institutes of Health, 16 Center Drive, Bethesda, MD 20892, USA; b Department of International Health, Johns Hopkins University, Baltimore, MD, 21205, USA; c Division of Infectious Diseases and International Health, University of Virginia, P.O. Box 801340, 345 Crispell Drive, Carter Harrison Building, Charlottesville, VA 22908, USA; d Aga Khan University, Department of Pediatrics and Child Health, Stadium Road, Karachi, Pakistan; e Asociaciόn Benéfica Proyectos de Informática, Salud, Medicina, y Agricultura (A.B. PRISMA), Ramirez Hurtado 622, Iquitos, Peru; f HIV/AIDS and Global Health Research Programme, University of Venda, Thohoyandou 0950, South Africa; g Department of Child Health, Institute of Medicine, Tribhuvan University, Katmandu, Nepal; h Centre for International Health, University of Bergen, P.O. Box 7800, 5020 Bergen, Norway; i Department of Gastrointestinal Sciences/Department of Community Health, Christian Medical College, Vellore, Tamil Nadu 632004, India; j Nutrition and Clinical Services Division, International Centre For Diarrhoeal Disease Research, Bangladesh (icddr,b), 68 Shaheed Tajuddin Ahmed Sarani, Mohakhali, Dhaka 1212, Bangladesh; k Haydom Lutheran Hospital, POB 9041, Haydom, Manyara Region, Tanzania; l Instituto de Biomedicina, Departamento de Fisiologia e Farmacologia, Faculdade de Medicina Federal University of Ceara, Rua Coronel Nunes de Melo, 1315, CEP: 60.430-270 - C.P. 3229 – Porangabussu, Fortaleza Ceará, Brazil; m Department of Environmental Science and Policy and the Duke Global Health Institute, Duke University, Durham, NC, USA; n Armed Forces Research Institute of Medical Sciences, Bangkok, Thailand

**Keywords:** MAL-ED, The Etiology, Risk Factors and Interactions of Enteric Infections and Malnutrition and the Consequences for Child Health and Development, EPI, Expanded Program on Immunization, BGD, Dhaka, Bangladesh, BRF, Fortaleza, Brazil, INV, Vellore, India, NEB, Bhaktapur, Nepal, PEL, Loreto, Peru, PKN, Naushero Feroze, Pakistan, SAV, Venda, South Africa, TZH, Haydom, Tanzania, BCG, Bacillus Calmette-Guérin, DPT, Diphtheria, Pertussis, and Tetanus, OPV, Oral Polio Vaccine, IPV, Inactivated Polio Vaccine, LTF, lost to follow up, WAMI, Water/sanitation, Assets, Maternal education and Income, DHS, Demographic Health Survey, WHO, World Health Organization, UNICEF, United Nations Children’s Fund, Public health, Vaccine coverage, Vaccine timing, EPI, Socioeconomic factors, Measles

## Abstract

**Background:**

Launched in 1974, the Expanded Program on Immunization (EPI) is estimated to prevent two-three million deaths annually from polio, diphtheria, tuberculosis, pertussis, measles, and tetanus. Additional lives could be saved through better understanding what influences adherence to the EPI schedule in specific settings.

**Methods:**

The Etiology, Risk Factors and Interactions of Enteric Infections and Malnutrition and the Consequences for Child Health and Development (MAL-ED) study followed cohorts in eight sites in South Asia, Africa, and South America and monitored vaccine receipt over the first two years of life for the children enrolled in the study. Vaccination histories were obtained monthly from vaccination cards, local clinic records and/or caregiver reports. Vaccination histories were compared against the prescribed EPI schedules for each country, and coverage rates were examined in relation to the timing of vaccination. The influence of socioeconomic factors on vaccine timing and coverage was also considered.

**Results:**

Coverage rates for EPI vaccines varied between sites and by type of vaccine; overall, coverage was highest in the Nepal and Bangladesh sites and lowest in the Tanzania and Brazil sites. Bacillus Calmette-Guérin coverage was high across all sites, 87–100%, whereas measles vaccination rates ranged widely, 73–100%. Significant delays between the scheduled administration age and actual vaccination date were present in all sites, especially for measles vaccine where less than 40% were administered on schedule. A range of socioeconomic factors were significantly associated with vaccination status in study children but these results were largely site-specific.

**Conclusions:**

Our findings highlight the need to improve measles vaccination rates and reduce delayed vaccination to achieve EPI targets related to the establishment of herd immunity and reduction in disease transmission.

## 1 Introduction

The Expanded Program on Immunization (EPI) was established to ensure that all children have access to and receive basic immunizations [1]. Vaccination schedules are designed to balance maximizing vaccine efficacy (i.e. targeting the ages for optimal immunological response) with high population coverage (i.e. leveraging frequent contacts with healthcare providers during the first months of life) to achieve high levels of vaccine effectiveness [2]. The EPI prevents an estimated two to three million child deaths annually; however, despite near global adoption of EPI recommendations, schedules and vaccination rates vary greatly by country. Steady increases in global vaccination rates since 1990 [3] suggest that the overall EPI target, that 90% of children in the world should be vaccinated with Bacillus Calmette-Guérin (BCG), 3rd dose of Diphtheria, Pertussis, and Tetanus (DPT3), 1st dose of measles vaccine (MCV1), and 3rd dose of Oral Polio Vaccine (OPV3) by 2020, is within reach [4]. This progress can be accelerated and significant disease burden reduced by better understanding the factors associated with vaccine coverage and timeliness. However, few studies have addressed the extent of delayed vaccination across multiple regions of the world [5], [6].

The Etiology, Risk Factors and Interactions of Enteric Infections and Malnutrition and the Consequences for Child Health and Development (MAL-ED) Study is a multi-site cohort study investigating the effects of undernutrition, gut function, and enteric disease on child development, growth, and vaccine response [7]. Children in the MAL-ED cohorts, located in Dhaka, Bangladesh (BGD), Fortaleza, Brazil (BRF), Vellore, India (INV), Bhaktapur, Nepal (NEB), Loreto, Peru (PEL), Naushero Feroze, Pakistan (PKN), Venda, South Africa (SAV), and Haydom, Tanzania (TZH), were followed for the first two years of life providing an opportunity to assess adherence to national EPI schedules in diverse settings [8], [9], [10], [11], [12], [13], [14], [15]. Here we describe vaccination coverage in the MAL-ED cohorts and examine adherence to country-specific EPI schedules. Additionally, we evaluate how socioeconomic and demographic factors are associated with vaccination and schedule adherence.

## 2 Methods

### 2.1 EPI schedule

Country-specific EPI schedules and vaccine information were collected by study personnel. For several countries, the EPI schedule was modified during the study period of 2009–2014; changes were accounted for where appropriate. Additionally, vaccine campaigns conducted throughout the study period were documented.

### 2.2 Child vaccination histories

Data collection methods have been previously described [16]. Briefly, the MAL-ED cohorts consisted of approximately 200 children per site followed from birth to 24 months of age [7]. The study was observational and vaccines were not administered by the study. A structured vaccine history questionnaire was administered during home visits on the monthly anniversary of the child’s birth (±2 days) to collect information on vaccine receipt. The mother/caregiver was asked to provide information on vaccinations since the previous visit, using the vaccine card issued by the health provider when possible or based on mother/caregiver recollection if no vaccine card was available. Additionally, a quarterly vaccine information form recorded vaccines received and date of administration based on the child’s vaccination card if present, clinical records or mother/caregiver’s best recollection; the source of the vaccination history was also noted and the records were furthermore used to confirm data from the monthly questionnaire. Approval to access health records of study children for vaccination information was received from local Internal Review Board. Extensive quality control activities were coordinated uniformly across all sites in real time. Vaccinations occurring outside the expected site-specific EPI schedule and vaccinations inconsistently reported on the two forms (monthly and quarterly) were reported back to the sites where study personnel made appropriate corrections after confirming the information with the source.

### 2.3 Analytical methods

Children with ⩾12 months of follow-up were included in the primary analysis. Depending on the country-specific schedule, regardless of vaccination age, children were considered fully vaccinated at 12 months of age with a minimum of 1 dose of BCG, 3 doses of DPT, 1 dose of measles vaccine, and 3–5 doses of Oral/Inactivated Polio Vaccine (OPV/IPV). For schedule adherence analyses, vaccinations were considered ‘on time’ if administered within 7 days of the scheduled time (14-day window). Per EPI recommendations, for vaccines with multiple doses, the scheduled interval between initial and subsequent doses was considered more important than the specific age at receipt of subsequent doses if the initial dose was off schedule. To assess bias in the sample due to drop outs, the proportion of children who adhered to the schedule prior to being lost to follow up (LTF) was estimated.

Student’s *t*-tests and tests to compare two proportions were used to compare fully vaccinated versus non-fully vaccinated children for overall socioeconomic status (the Water/sanitation, Assets, Maternal education and Income [WAMI] index) [17], and factors including household income in US dollars, maternal age, years of maternal education, number of siblings in the household, sex, whether the child was first born, and place of delivery. Proportions tests were used to examine timeliness of vaccination; age at the first dose of BCG, DPT, OPV, or measles were indicators for schedule adherence. p-Values equal to or below 0.10 were considered significant. All analyses were performed using STATA version 13 (StataCorp LP. College Station, TX).

## 3 Results

The MAL-ED cohorts were selected to represent a broad range of low and middle income country settings including a mix of urban and rural locations where malnutrition and enteric disease burden were high [8], [9], [10], [11], [12], [13], [14], [15]. The median monthly household income across all sites was $113 (range $0–1648/month) ranging from $14 in TZH to $347 in BRF and the median maternal age was 26 years (range 14–49 years) while the median maternal education was 7 years (range 0–20 years) (Supplementary Table 1).

### 3.1 EPI schedule

Country-specific EPI schedules varied between sites (Table 1). DPT, Hepatitis B (HepB), and Haemophilus influenzae type b (Hib) vaccine were administered simultaneously in a pentavalent formulation in most MAL-ED sites. In BRF, during the early study period, tetravalent vaccine (DPT + Hib) was used with HepB administered separately before pentavalent vaccine introduction in July 2012. In INV, Hib vaccine was introduced with the switch to pentavalent vaccine in December 2011. In SAV, the pentavalent vaccine utilized contained DPT, Hib, and IPV with HepB administered separately. In PEL, HepB was given at birth before later administration of pentavalent vaccine. SAV was the only site using a combined OPV/IPV schedule in their polio vaccine program, which was introduced in 2009 [18].

Substantial heterogeneity between the design and infrastructure of vaccine delivery systems was observed (Table 2). Sites offered all EPI vaccines free of charge at local hospitals or health clinics, with the cost covered by government or non-governmental organizations. The timing and frequency of vaccine availability was variable between sites and vaccines and may have influenced timeliness of administration. However, in all sites except for BRF and SAV, all vaccines were offered at least weekly. Community-wide vaccine campaigns occurred in all sites. OPV was the most frequent component of campaigns, which occurred in all sites except Tanzania. Pakistan and India frequently employed this strategy with 29 and 10 campaigns recorded during the study period, respectively. Children received up to 19 doses in PKN and up to 11 doses in INV by age 24 months (Supplementary Fig. 1).

### 3.2 MAL-ED vaccine coverage

The primary source of vaccine histories was vaccination cards; 80% of DPT1 records were obtained from vaccine cards, 6% were verbal caregiver records, while 12% came from clinical records. There were differences between the sites in vaccination history sources; 100% of vaccination histories in BGD, BRF, and NEB were obtained from vaccine cards compared to 5% in PKN; where the majority of information (83%) came from clinical records.

Vaccination rates varied greatly by site and vaccine (Table 3 and Supplementary Table 2). NEB had complete coverage for all vaccines. The low 15% coverage rate in TZH was driven by OPV; 22% of children were fully vaccinated against polio whereas other vaccines reached >70%. BGD had homogeneously high coverage for all vaccines in contrast to BRF where close to 100% of children received BCG while only 73% received measles vaccine. Sites using pentavalent vaccine throughout the study period reached coverage ranging from 73% in TZH, 89% in PKN, 96% in BGD, to 100% in NEB. Hib vaccine was introduced in INV after most of the cohort had surpassed appropriate vaccination age resulting in only 20% coverage. The global EPI target of fully vaccinated rates of >90% for all children, was reached in only two sites (BGD and NEB), though many individual vaccines had coverage levels >90% at most sites. Within the MAL-ED cohort, 75% of all children were fully vaccinated by age 24 months. Children LTF before 12 months of age had lower vaccine coverage than children meeting the inclusion criteria (Supplementary Table 3).

### 3.3 EPI schedule adherence

**Fig. 1:**
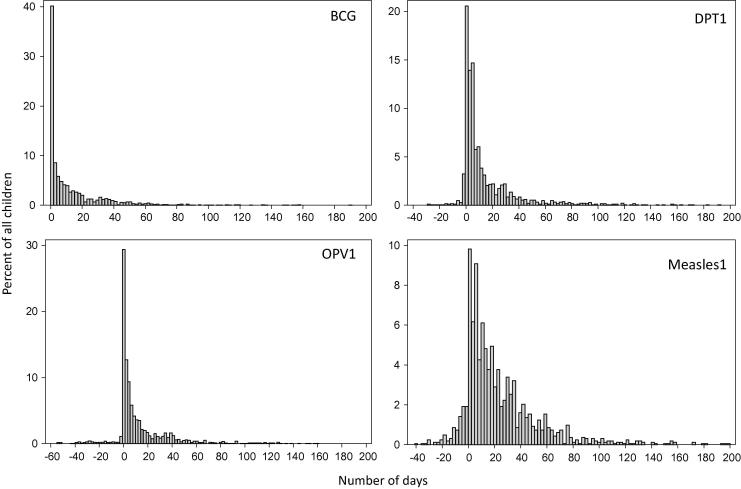
Schedule adherence. Number of days between scheduled and actual vaccination day for the first dose of BCG, DPT, OPV, and measles for all children. BCG1: first dose of BCG; DPT1: first dose of DPT; Measles1: first dose of measles.

**Fig. 2:**
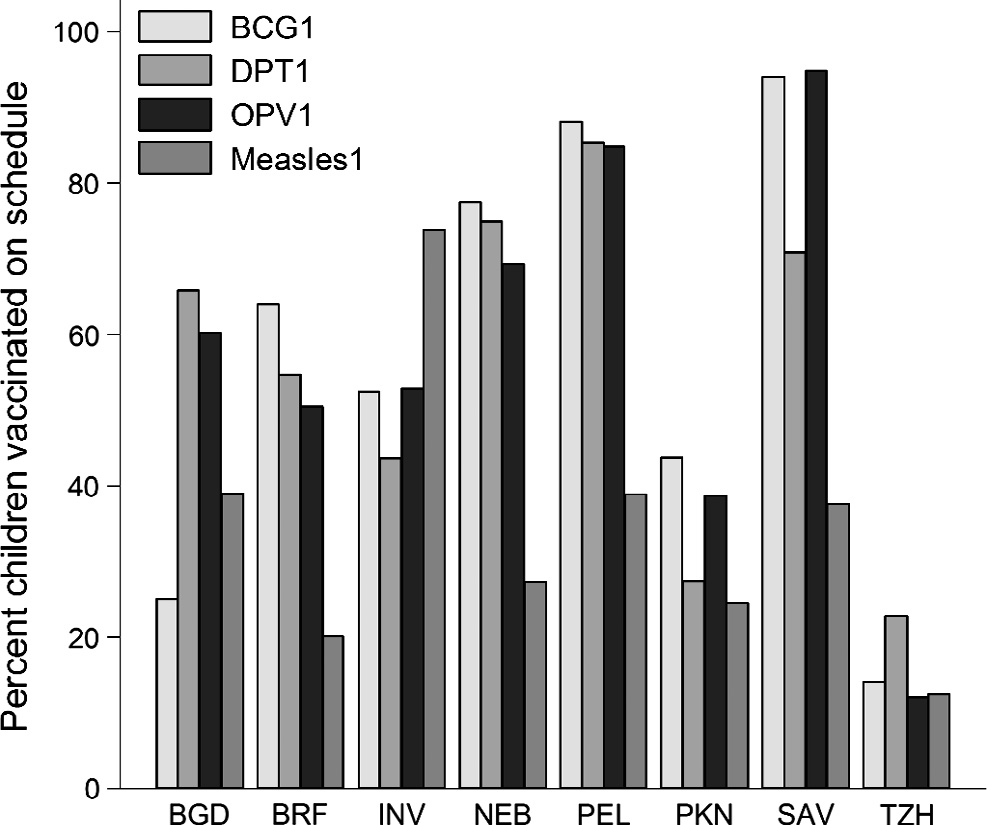
Schedule adherence by vaccine. Percent children vaccinated on schedule (within 7 days of the EPI scheduled date) by vaccine by site. BCG1: first dose of BCG; DPT1: first dose of DPT; Measles1: first dose of measles; BGD: Dhaka, Bangladesh; BRF: Fortaleza, Brazil; INV: Vellore, India; BGD: Bhaktapur, Nepal; PEL: Loreto, Peru; PKN: Naushero Feroze, Pakistan; SAV: Venda, South Africa; TZH: Haydom, Tanzania.

Administration of vaccines was delayed at all sites to a variable degree (Fig. 1). BCG is scheduled at birth, and the majority received the vaccine on time, the median was 4 days ranging from 1 day in NEB, PEL, and SAV to 38 days in TZH. The medians for DPT1 and OPV1 were 5.56 and 4 days, respectively. Age at measles vaccination ranged broadly with the majority administered within 60 days of the scheduled date with a median of 16 days ranging from 10.5 days in SAV to 36 days in INV. Adherence to the EPI schedule varied considerably between sites and vaccines (Fig. 2). Generally, BCG was more likely to be given on time, although there was significant site-to-site variability (14% in TZH and 94% in SAV). DPT1 was administered on time in >50% of children in BGD, BRF, NEB, PEL, and SAV compared to 23% in TZH. A similar pattern emerged in the timing of OPV1 ranging from 12% in TZH to 95% in SAV but with PKN and TZH as the only sites with <50% adherence. Measles vaccine had low schedule adherence in most sites ranging from 12% of children in TZH to 39% in BGD and PEL. In INV, measles vaccine was administered on time in 74% of children; however instead of a fixed vaccination time, India has a window from 9 to 12 months hence a larger window for ‘on time’ vaccination. In most cases when an initial vaccine was delayed, successive doses were then appropriately adjusted to maintain EPI-recommended intervals between doses (data not shown).

### 3.4 Influence of socioeconomic factors on vaccination status and schedule adherence

Associations between socioeconomic factors and achievement of complete and on-time vaccination were largely site-specific. The exception was birth setting, which was associated with the likelihood of a child being fully vaccinated and/or the likelihood of vaccine schedule adherence in every site except SAV (Table 4). The majority of study children were born in a hospital (private or public) or health clinic and only 3 sites had >10% of children born in the home; in PKN, TZH, and BGD 59%, 50%, and 30% of children were born at home, respectively. In TZH, only 4% of children born at home received the BCG and OPV at birth whereas 24% and 20% of the children born in a hospital facility received it on time, respectively. In PKN and BGD, only 85% of children born at home were fully vaccinated compared to 92% and 97%, respectively of the children born at a health facility. In INV, fully-vaccinated children were more likely to live in households with fewer other children, more highly-educated mothers and higher WAMI scores, than their non-fully vaccinated counterparts (Table 5). More years of maternal education and higher WAMI index were significantly associated with full vaccination in PKN. Maternal age and household income were not significantly associated with complete vaccination in any site; however, higher household income tended to associate with full vaccination.

In INV, fewer total children in the household, higher maternal education, and higher WAMI index were associated with greater vaccine schedule adherence while in other sites few of these relationships were present (Supplementary Table 4). Sex was inconsistently associated with vaccination rates and schedule adherence: in PKN, females were more likely to receive measles vaccine on time and in BGD, females were more likely to receive BCG on time. In INV, first born children were more likely to be fully vaccinated and to receive measles vaccine on time (Supplementary Table 5).

## 4 Discussion

The MAL-ED study included intensive, prospective surveillance of vaccinations obtained from local health sources in eight communities, creating an opportunity for detailed analysis of vaccine coverage and timeliness under circumstances resembling real-world conditions [7]. Measles coverage was lowest among the vaccines analyzed in this study with only three sites having rates above the 95% required for development of herd immunity. This low coverage is concerning as measles incidence has been increasing worldwide in recent years [19]. Globally, coverage is 84%, well below the threshold to stop transmission, as is the overall 89% coverage in MAL-ED and in TZH, where coverage is low at 76% we saw a measles outbreak during 2012 with 68 cases admitted to the hospital. These low rates suggest the goal of measles elimination is likely to remain elusive without enhanced efforts to improve measles vaccine coverage [20]. Recent experimental and epidemiological studies [21], [22] provide complementary evidence that measles infection reduces population immunity to other infectious diseases for up to three years. The persistence of low measles vaccine coverage represents a missed opportunity to not only prevent mortality and morbidity from measles, but also other childhood infections.

All MAL-ED sites participated in vaccine campaigns. Six sites utilized measles vaccine campaigns; the two sites not doing so were those with the lowest coverage, BRF and INV, suggesting that measles campaigns might help boost overall coverage. Although, TZH did have a measles campaign, increases in vaccination during this period did not occur—suggesting that more promotion is necessary to fully realize the mop-up potential of a campaign. Polio campaigns were most frequent and utilized extensively in Pakistan and India where polio is still a concern. Consequently, children in these sites received a large number of extra doses. A study in India found that the intense focus on polio eradication did not adversely affect coverage for other routine vaccinations; instead, DPT3 coverage increased between 2008 and 2011 [23]. Similarly, vaccine coverage in the PKN and INV sites did not suffer compared to coverage in other MAL-ED cohorts. The absence of OPV campaigns in MAL-ED’s TZH cohort and the extremely low 22% coverage rate suggest that routine administration of OPV (and the planned 2016 introduction of the OPV-IPV combination) may need to be enhanced by campaign efforts to sufficiently support conditions for global polio eradication [24]. Generally, the positive effect of campaigns related to vaccine coverage rates suggests that these campaigns effectively boosted immunization rates and are a useful tool to reach the EPI target of 90% coverage. Similarly, more campaigns offering multiple EPI vaccines would increase the accessibility of all vaccines.

The MAL-ED study obtained information on vaccination history from a variety of sources. The coverage data primarily came from vaccination cards but there was heterogeneity in information sources within and between sites. The low retention of vaccination cards found in PKN was consistent with previous findings in Pakistan [25]. A recent review of the reliability of vaccine data found that data from vaccine cards likely underestimate true coverage whereas data from recall and medical provider sources tend to be overestimates [26]. However, based on frequent surveillance and communication with site personnel, we consider the MAL-ED estimates robust.

Compared to country-specific coverage rates found by the Demographic Health Survey (DHS), World Health Organization (WHO) and United Nations Children’s Fund (UNICEF), for individual EPI vaccines the MAL-ED cohort rates were higher for all sites except Tanzania where coverage was lower across all vaccines (Supplementary Table 6). According to WHO/UNICEF estimates, four of the countries containing MAL-ED sites had coverage >90% for DPT, OPV, and measles; whereas estimates were lower than 90% in South Africa, India, Peru, and Pakistan [27]. The WHO/UNICEF estimates are primarily based on administrative data from vaccination service providers while DHS are a cross-sectional view of vaccine coverage making a direct comparison unreliable. Despite this study finding mostly higher coverage rates than national estimates, it is worth noting that concerns remain about the overestimation of vaccine coverage in countries where immunization funding relies on a performance-based system. The discrepancies between such findings emphasize the need for higher quality and more systematic monitoring of such indicators to better inform and improve global vaccination efforts [28].

For many low resource countries, the convenience of the EPI schedule is as important as timeliness of vaccination for development of herd immunity and for reduction in disease burden [6]. In the MAL-ED cohort, BCG coverage was highest with >97% in 7 sites. BCG was also most likely to be administered on schedule, largely due to the fact that it is given at birth. When delivery occurs in a health facility, the child is easily accessible for on-time vaccine administration. In 3 sites there were significant differences in the timeliness of BCG administration depending on birth location. In TZH, timeliness of BCG was low in hospitals as well as home births and that could be attributed to problems with supply. However, we did not see large gaps in the vaccination date and since many children did not receive the vaccine until 1, 2 or 3 months of age there are likely other reasons in play as well. Later vaccines require the caregiver to bring the child to immunization facilities, increasing the risk of a delayed or missed appointment. Measles vaccine was most likely to be delayed, possibly because it is given later in life (9–12 months of age) and often given as the only vaccination at that time. This was also shown in a South African study where timeliness and coverage were lower for later vaccinations and in poorer populations [29]. In NEB, where coverage was 100% for all vaccines but timeliness was suboptimal, the delay was attributed to a variety of reasons including: less focus on timely vaccination as opposed to getting vaccinated, the frequent travel necessary to accommodate the EPI schedule, and a tendency for parents to postpone vaccination during mild illness or discomfort of the child. Similarly, the low coverage and timeliness in TZH was attributed in part to rural surroundings of the TZH field site, along with lack of communication between health facilities and the population. These are areas that could be improved with increased focus on communication from the vaccine providers and educational materials to the population regarding the importance of getting vaccinations and getting them on schedule. In addition to examining a ±7-day window around the scheduled day as a marker for timeliness, we also tested a ±14-day window for sensitivity (data not shown). We decided to use the ±7-day window, as the broader window did not change the associations found and therefore would not help to improve the estimates of timeliness.

We evaluated correlations between socioeconomic and demographic factors and coverage and schedule adherence; the diversity of findings between sites reflects the culture- and country-dependent nature of the SES factors measured. A high percentage of cohort children had high coverage for individual vaccines but in the sites with the lowest coverage, TZH and BRF, there were no factors that stood out as associated with lower coverage. A Brazilian study found that birth order and higher household income were associated with higher risk of incomplete vaccination [30]. Although we were not able to confirm those results we did find that being a first born child was a predictor for on time vaccination with BCG and DPT. A Tanzanian study found that maternal education was associated with not being vaccinated while poverty and maternal education were associated with delayed vaccination [31]. None of these relationships were seen in the MAL-ED cohort, however the small sample size and similar poverty level throughout the cohort could explain the lack of associations. Lastly, indications from several of the MAL-ED sites suggest that travel distance to health clinics and frequency were inhibitory for timely and complete vaccination status, however we were not able to investigate this further in this study.

### 4.1 Limitations

We examined vaccine coverage in children age 0–24 months; this observation period may lead to an underestimate of the total vaccination coverage in the life of a child. A study of vaccine coverage in low- and middle-income countries found increased coverage when including children up to age 36 months allowing for late catch-up vaccination. Measles vaccine coverage increased from 54% at 12 months to 82% at 36 months indicating that many children were vaccinated at older ages [6]. Children LTF before 12 months of age had lower vaccine coverage while under observation compared to children remaining in the study beyond 12 months. This could lead to a potential overestimation of the vaccine coverage in the cohort while also indicating that our study population might have higher vaccination rates and adherence compared to the general population. Furthermore, the intensive surveillance could have influenced coverage as caregivers may have been more likely to pursue vaccination; however, we found that only 2 sites reached the EPI target of >90% coverage for all vaccines among all cohort children.

## 5 Conclusion

Childhood vaccinations are an essential public health tool to prevent serious disease in young children and optimal effectiveness requires vaccine administration according to the EPI schedule. We found that despite high vaccine coverage in most MAL-ED sites, vaccine administration was frequently delayed, especially measles vaccine. Delayed vaccination has consequences for the development of herd immunity and disease transmission; the continued occurrence of measles outbreaks likely results from such subpar timeliness and coverage and prolongs needless mortality and morbidity from measles and other childhood infections. The diversity between sites suggests that country-specific interventions are needed to meet EPI targets and better measure target indicators.

## Contributors

CH performed statistical analysis, interpreted results and drafted the manuscript with input from all authors. JCS, GL, JAPM, SLK, CJM, WP interpreted results and edited the manuscript. SLK, CJM, PB, ERM, MAM planned and supervised the project. AA, MPO, PB, RC, SB, VRM, DM, MM, ERM, EN, CA participated in data collection and provided site expertise. All authors reviewed and approved the manuscript.

## Conflict of interest

None

## Funding source

The Etiology, Risk Factors and Interactions of Enteric Infections and Malnutrition and the Consequences for Child Health and Development Project (MAL-ED) is carried out as a collaborative project supported by the Bill & Melinda Gates Foundation, the Foundation for the NIH, and the National Institutes of Health, Fogarty International Center.

## Figures and Tables

**Table 1:** EPI vaccine schedule for selected vaccines in MAL-ED countries. Several changes to the schedules occurred throughout the study period; the schedule reflects the schedule at the end of data collection period**.** BCG: Bacillus Calmette-Guiren; OPV: Oral Polio Vaccine; IPV: Inactivated Polio Vaccine; DPT: Diphtheria, Pertussis, Tetanus; HEPB: Hepatitis B; Hib: Heaemophilus influenzae type b; w: weeks; m: months.

Vaccine/# doses	Bangladesh	Brazil	India	Nepal	Peru	Pakistan	South Africa	Tanzania
	BGD	BRF	INV	NEB	PEL	PKN	SAV	TZH
BCG	Birth	Birth	Birth	Birth	Birth	Birth	Birth	Birth

OPV1	6w	2m	Birth	6w	2m	Birth	Birth	Birth
OPV2	10w	4m	6w	10w	4m	6w	6w	1m
OPV3	14w	6m	10w	14w	6m	10w		2m
OPV4	9m	15m	14w			14w		3m
OPV5			16–24m					

IPV1							6w	
IPV2							10w	
IPV3							14w	
IPV4							18m	
DPT1	6w	2m	6w	6w	2m	6w	6w	1m
DPT2	10w	4m	10w	10w	4m	10w	10w	2m
DPT3	14w	6m	14w	14w	6m	14w	14w	3m
DPT4		15m	16–24m		18m		18m	

HEPB1	6w	Birth	6w	6w	Birth	6w	6w	1m
HEPB2	10w		10w	10w	2m	10w	10w	2m
HEPB3	14w	1m	14w	14w	4m	14w	14w	3m
HEPB4		6m			6m			

Hib1	6w	2m	6w	6w	2m	6w	6w	1m
Hib2	10w	4m	10w	10w	4m	10w	10w	2m
Hib3	14w	6m	14w	14w	6m	14w	14w	3m
Hib4					18m		18m	

Measles1	9m	12m	9–12m	9m	12m	9m	9m	9m
Measles2	15–18m	15m	(12–15m)	(15m)^a^		15m	18m	

a Optional with payment. Optional vaccines in parentheses.

**Table 2:** Source, schedule, distribution, and availability of vaccines by site. Information collected from the site personnel. BGD: Dhaka, Bangladesh; BRF: Fortaleza, Brazil; INV: Vellore, India; NEB: Bhaktapur, Nepal; PEL: Loreto, Peru; PKN: Naushero Feroze, Pakistan; SAV: Venda, South Africa; TZH: Haydom, Tanzania.

	BGD	BRF	INV	NEB	PEL	PKN	SAV	TZH
**Distributors of vaccine**	Public and NGOs	Public	Public and private	Public and private	Public	Public	Government outsource to private companies	Public

**Location of distribution**	NGO based community outposts; public hospitals	Public health centers; public hospitals	Primary Health Centers; health clinics and mobile units run by the Govt.	Local hospitals and health centers	Public Hospitals, Health Centers, and Health Posts (not private clinics)	Primary health centers and public hospitals	Health clinics and hospitals	Local hospitals and health clinics

**Schedule for distribution**	Public hospitals run vaccination activities throughout the year	Beginning of each month	Vaccines are administered on a scheduled day every week; 4 times a month	3 days a week at the local hospital and vaccination clinics on Saturdays in one community	All days except Sundays, from around 7am to 1 pm. 1 day a week to distribute a particular vaccine	Measles and BCG available once a week, other vaccines available all days except Sunday from 9 am to 2 pm	Every two weeks	Daily in hospitals and health clinics and monthly in mobile clinics

**Availability of vaccination**	Widely available	Widely available, when limited prioritization by age, younger to older	Widely available, restrictions based on geography and location	Widely available through hospitals and clinics	Widely available	Widely available throughout the community and year around	Widely available	Widely available

**Cost to families**	All EPI vaccines are free	All EPI vaccines are free	All EPI vaccine are free	All EPI vaccines are free	All EPI vaccines are free	All EPI vaccines are free	All EPI vaccines are free	All EPI vaccines are free

**Vaccine campaigns**	4 or more each year through inter agency Co-ordination Committee meeting. They use all types of media for campaigning	Campaigns are national and is programmed by the ministry of health. Requested by a municipal can be made	Pulse Polio campaigns are held by the Govt. twice a year in January & February, where OPV is administered to all <5 years	No specific campaigns for EPI regular vaccines. OPV campaigns are offered through mobile vaccination clinics	Few campaigns run from the health centers free of cost to the families^a^	OPV national immunization days twice a year. Supplementary vaccination days frequently throughout the year. Measles and Tetanus campaign through special initiatives	Campaigns are organized by mobile clinics and specific locations like schools and local Chiefs’ kraals	Few campaigns run from mobile units. Free of cost

**No. Vaccine campaigns**	7 OPV	7 OPV	10 OPV	11 OPV	2 OPV	29 OPV^b^	4 OPV	1 MEA
	1 MEA			1 MEA	1 DPT	3 MEA	4 MEA	All Vaccines^c^
					1 MMR			

a This is done when a vaccine lot is near expiration, about once a year. The health post nurses are paid extra to go from house to house looking for children <5 years; if they are unable to show record of vaccination, eligible children are vaccinated.

b PKN information only up to August 2013.

c TZH had a campaign to cover all vaccines in April 2013.

**Table 3:** Number of children enrolled, number of children with 12 months of follow-up (%), and percent of children vaccinated fully according to schedule by age 12 months (95% confidence intervals). Fully vaccinated ALL includes BCG, DPT, OPV/IPV, and measles. BGD: Dhaka, Bangladesh; BRF: Fortaleza, Brazil; INV: Vellore, India; NEB: Bhaktapur, Nepal; PEL: Loreto, Peru; PKN: Naushero Feroze, Pakistan; SAV: Venda, South Africa; TZH: Haydom, Tanzania.

	**Enrolled**	**⩾12m follow-up (%)**	**Complete vaccination BCG**	**Complete vaccination DPT**	**Complete vaccination OPV/IPV**	**Complete vaccination Measles**	**Fully vaccinated ALL**	**Complete vaccination HepB**	**Complete vaccination Hib**
BGD	265	231 (87)	100	96.1 (93.6–98.6)	97.8 (95.9–99.7)	96.5 (94.2–98.9)	93.9 (90.8–97.0)	96.1 (93.6–98.6)	96.1 (93.6–98.6)

BRF	233	194 (83)	100	75.3 (69.1–81.4)	86.1 (81.2–91.0)	73.2 (66.9–79.5)	59.3 (52.3–66.2)	77.8 (71.9–83.7)	75.3 (69.1–81.4)

INV	251	229 (91)	99.1 (97.9–100.3)	82.5 (77.6–87.5)	91.7 (88.1–95.3)	86 (81.5–90.5)	78.2 (72.8–83.6)	72 (66.2–77.9)	19.6 (14.5–24.8)

NEB	240	231 (96)	100	100	100	100	100	100	100

PEL	303	244 (80)	98.8 (97.4–100.2)	96.7 (94.5–99.0)	97.1 (95.0–99.2)	90.2 (86.4–93.9)	86.1 (81.7–90.4)	90.2 (86.4–93.9)	96.7 (94.5–99.0)

PKN	277	256 (92)	98.8 (97.5–100.1)	89 (85.2–92.9)	100	99.6 (98.8–100.4)	88.3 (84.3–92.2)	89.1 (85.2–92.9)	89.1 (85.2–92.9)

SAV	314	253 (81)	96.8 (94.7–99.0)	82.6 (77.9–87.3)	78.3 (73.1–83.4)	89.3 (85.5–93.2)	72.7 (67.2–78.2)	87.3 (83.2–91.5)	82.6 (77.9–87.3)

TZH	262	233 (89)	87.1 (82.8–91.5)	72.1 (66.3–77.9)	21.9 (16.5–27.2)	76.4 (70.9–81.9)	15.4 (10.8–20.1)	72.1 (66.3–77.9)	72.1 (66.3–77.9)

Total	2145	1871 (87)	97.5 (96.8–98.2)	87.1 (85.5–88.6)	84.2 (82.6–85.9)	89.4 (88.0–90.8)	74.7 (72.7–76.7)	85.8 (84.2–87.4)	79.4 (77.5–81.2)

**Table 4:** Comparison of proportions (95% confidence intervals) of children born in the hospital or in the home by MAL-ED site with regards to schedule adherence and full vaccination status. Hospital includes public and private hospital and health clinics. BCG1: first dose of BCG; DPT1: first dose of DPT; Measles1: first dose of measles; fully vaccinated: received ⩾1 BCG, ⩾3 DPT, ⩾3–5 Polio, and ⩾1 measles; BGD: Dhaka, Bangladesh; BRF: Fortaleza, Brazil; INV: Vellore, India; NEB: Bhaktapur, Nepal; PEL: Loreto, Peru; PKN: Naushero Feroze, Pakistan; SAV: Venda, South Africa; TZH: Haydom, Tanzania.

	**Place of birth**	**N**	**BCG1 On time**	**DPT1 On time**	**OPV1 On time**	**Fully vaccinated**
BGD	Hospital	159	0.23 (0.17–0.30)	0.63 (0.55–0.70)	0.57 (0.50–0.65)	**0.97 (0.95**–**1.00)**
	Home	69	0.30 (0.20–0.41)	0.72 (0.62–0.83)	0.67 (0.56–0.78)	**0.85 (0.77**–**0.94)**
	P-value		0.25	0.16	0.18	**<0.01**

BRF	Hospital	185	0.63 (0.56–0.70)	**0.57 (0.50**–**0.64)**	**0.52 (0.45**–**0.60)**	**0.61 (0.54**–**0.68)**
	Home	5	0.60 (0.17–1.03)	**0.00**	**0.00**	**0.20 (−0.15 to 0.55)**
	P-value		0.88	**0.01**	**0.02**	**0.06**

INV	Hospital	222	0.53 (0.46–0.59)	0.44 (0.38–0.51)	0.53 (0.47–0.90)	**0.79 (0.73**–**0.84)**
	Home	6	0.50 (0.10–0.90)	0.17 (−0.13 to 0.46)	0.50 (0.10–0.90)	**0.50 (0.10**–**0.90)**
	P-value		0.90	0.18	0.88	**0.09**

NEB	Hospital	225	**0.80 (0.74**–**0.85)**	**0.76 (0.70**–**0.82)**	**0.70 (0.64**–**0.76)**	a
	Home	6	**0.00**	**0.33 (−0.04 to 0.71)**	**0.33 (−0.04 to 0.71)**	
	P-value		**<0.01**	**0.02**	**0.05**	

PEL	Hospital	230	**0.90 (0.87**–**0.94)**	0.86 (0.81–0.90)	0.85 (0.81–0.90)	0.86 (0.82–0.91)
	Home	14	**0.50 (0.24**–**0.76)**	0.79 (0.57–1.00)	0.79 (0.57–1.00)	0.86 (0.67–1.04)
	P-value		**<0.01**	0.47	0.50	0.97

PKN	Hospital	106	0.46 (0.37–0.56)	0.27 (0.19–0.36)	0.38 (0.28–0.47)	**0.92 (0.87**–**0.97)**
	Home	150	0.42 (0.34–0.50)	0.27 (0.20–0.34)	0.39 (0.31–0.47)	**0.85 (0.80**–**0.91)**
	P-value		0.50	1.00	0.80	**0.08**

SAV	Hospital	220	0.94 (0.91–0.97)	0.71 (0.65–0.77)	0.95 (0.92–0.98)	0.73 (0.67–0.79)
	Home	2	1.00	1.00	1.00	1.00
	P-value		0.72	0.37	0.75	0.39

TZH	Hospital	117	**0.24 (0.16**–**0.32)**	0.21 (0.14–0.29)	**0.20 (0.12**–**0.27)**	0.16 (0.10–0.23)
	Home	116	**0.04 (0.01**–**0.08)**	0.24 (0.16–0.32)	**0.04 (0.01**–**0.08)**	0.15 (0.08–0.21)
	P-value		**<0.01**	0.61	**<0.01**	0.74

a All children in NEB were fully vaccinated. Bold numbers indicate a significant finding: p-value <0.10.

**Table 5:** Socioeconomic characteristics for fully (+Full Vax) and not-fully (-Full Vax) vaccinated children (fully vaccinated defined as ⩾ 1 BCG,⩾3 DPT,⩾3–5Polio, and ⩾ 1 Measles doses) analyzed by comparison of the proportions (95% confidence intervals) of children fully or not fully vaccinated. BGD: Dhaka, Bangladesh; BRF: Fortaleza, Brazil; INV: Vellore, India; NEB: Bhaktapur, Nepal; PEL: Loreto, Peru; PKN: Naushero Feroze, Pakistan; SAV: Venda, South Africa; TZH: Haydom, Tanzania.

	***t*****-test**	**N**^a^	**Number of children in household**	**Maternal age**	**Years of maternal education**	**WAMI**	**Household income ($US dollars)**
BGD	−Full Vax	13–14	2.15 (1.46–2.84)	24.2 (21.0–27.4)	4.5 (2.8–6.2)	0.55 (0.47–0.63)	131 (95–168)
	+Full Vax	204–217	1.91 (1.76–2.05)	25.0 (24.3–25.6)	4.6 (4.2–5.0)	0.53 (0.51–0.54)	127 (113–141)
	P-value		0.41	0.60	0.89	0.62	0.89

BRF	−Full Vax	79	2.48 (2.13–2.83)	25.4 (24.3–26.5)	9.3 (8.7–9.8)	0.82 (0.8–0.84)	343 (311–375)
	+Full Vax	115	2.23 (1.99–2.47)	25.7 (24.6–26.8)	8.9 (8.4–9.5)	0.82 (0.9–0.84)	360 (333–387)
	P-value		0.21	0.71	0.40	0.92	0.42

INV	−Full Vax	49–50	**2.69 (2.22**–**3.17)**	25.2 (23.8–26.6)	**5.8 (4.7**–**6.9)**	**0.38 (0.34**–**0.42)**	71 (60–83)
	+Full Vax	175–176	**2.00 (1.83**–**2.17)**	24.2 (23.6–24.8)	**7.2 (6.7–7.4)**	**0.47 (0.45**–**0.49)**	77 (69–84)
	P-value		**<0.01**	0.13	**0.02**	**<0.01**	0.49
NEB^b^	−Full Vax	0	–	–	–	–	–
	+Full Vax	231					
	P-value						

PEL	−Full Vax	34	2.47 (1.83–3.11)	23.9 (21.7–26.0)	7.2 (6.2–8.2)	0.51 (0.45–0.56)	137 (104–169)
	+Full Vax	209–210	2.43 (2.20–2.65)	24.8 (24.0–25.6)	7.8 (7.4–8.1)	0.54 (0.53–0.56)	138 (128–148)
	P-value		0.88	0.42	0.30	0.13	0.91

PKN	−Full Vax	30	3.43 (2.64–4.23)	28.6 (26.6–30.6)	**1.7 (0.6**–**2.8)**	**0.41 (0.34**–**0.47)**	141 (101–181)
	+Full Vax	225–226	3.22 (2.94–3.49)	28.5 (27.7–29.3)	**3.3 (2.7**–**3.8)**	**0.50 (0.47**–**0.52)**	181 (162–199)
	P-value		0.60	0.93	**0.04**	**0.01**	0.14

SAV	−Full Vax	53–69	**2.70 (2.23**–**3.16)**	27.9 (25.9–30.0)	9.9 (9.4–10.5)	0.73 (0.69–0.77)	223 (182–263)
	+Full Vax	126–184	**2.26 (2.04**–**2.49)**	27.6 (26.4–28.9)	10.2 (9.9–10.4)	0.76 (0.74–0.78)	263 (219–307)
	P-value		**0.06**	0.81	0.36	0.124	0.27

TZH	−Full Vax	191–195	4.03 (3.69–4.37)	29.4 (28.5–30.3)	5.1 (4.7–5.5)	0.21 (0.20–0.23)	27 (23–32)
	+Full Vax	36	4.03 (3.18–4.87)	29.9 (27.6–32.3)	4.9 (3.9–5.8)	0.19 (0.15–0.23)	36 (18–54)
	P-value		0.99	0.66	0.62	0.25	0.18

a N varies between variables analyzed.

b All children in NEB were fully vaccinated. Bold numbers indicate a significant finding: p-value <0.10.

## Appendix A Supplementary material

Supplementary data 1 Supplementary file contains Supplementary Fig. 1 and Supplementary Tables 1–6.

Supplementary data 2 MAL-ED Network Investigators – complete list.
