# Supplementary material for: Vaccine coverage and adherence to EPI schedules in eight resource poor settings in the MAL-ED cohort study
Source: Vaccine. 2017 Jan 11;35(3):443–51. doi: 10.1016/j.vaccine.2016.11.075 (PMC5244255; doi:10.1016/j.vaccine.2016.11.075)
Supplement: Supplementary data 1 — Supplementary file contains Supplementary Fig. 1 and Supplementary Tables 1–6. [file mmc1.doc]

**Vaccine coverage and adherence to EPI schedules in eight resource poor settings in the MAL-ED cohort study**

Supplementary material:

Supplementary Tables 1-6

Supplemental Figure 1

|  | **BGD** | **BRF** | **INV** | **NEB** | **PEL** | **PKN** | **SAV** | **TZH** | **Total** |
| --- | --- | --- | --- | --- | --- | --- | --- | --- | --- |
| **Monthly income ($)** | 102  (36-924) | 347  (0-901) | 63  (9-357) | 138  (14-932) | 129  (4-429) | 134  (23-812) | 185  (34-1648) | 14  (1-240) | 113  (0-1648) |
| **Maternal education (years)** | 5  (0-14) | 9  (0-15) | 8  (0-18) | 9  (0-17) | 8  (0-20) | 0  (0-16) | 11  (3-15) | 7  (0-10) | 7  (0-20) |
| **Maternal age (years)** | 25  (17-40) | 25  (16-43) | 24  (17-40) | 27  (18-36) | 24  (14-42) | 27  (18-49) | 27  (16-45) | 29  (17-48) | 26  (14-49) |
| **Live births** | 2  (1-8) | 2  (1-8) | 2  (1-11) | 2  (1-5) | 2  (1-11) | 3  (1-17) | 2  (1-8) | 4  (1-16) | 2  (1-17) |

**Supplementary Table 1**. Socio-economic factors in the MAL-ED cohort. The number indicate the median while the parenthesis shows the ranges. BGD: Dhaka, Bangladesh; BRF: Fortaleza, Brazil; INV: Vellore, India; NEB: Bhaktapur, Nepal; PEL: Loreto, Peru; PKN: Naushero Feroze, Pakistan; SAV: Venda, South Africa; TZH: Haydom, Tanzania.

|  | **# doses** | **BGD**  **N=231** | **BRF**  **N=194** | **INV**  **N=229** | **NEB**  **N=231** | **PEL**  **N=244** | **PKN**  **N=256** | **SAV**  **N=253** | **TZH**  **N=233** |
| --- | --- | --- | --- | --- | --- | --- | --- | --- | --- |
| **BCG** | 0  **1**  **2** | **100.0** | **98.45**  **1.55** | 0.87  **99.13** | **100.0** | 1.23  **98.77** | 1.17  **98.83** | 3.16  **96.84** | 12.88  **81.97**  **5.15** |
| **DPT** | 0  1  2  **3**  **4**  **5** | 0.43  0.43  2.60  **96.54** | 1.55  3.61  14.43  **44.85**  **35.57** | 1.75  4.37  5.24  **24.02**  **64.63** | **100.0** | 1.23  **35.25**  **55.74**  **7.79** | 4.69  6.25  **89.06** | 0.40  3.16  8.30  **38.74**  **49.01**  **0.40** | 1.72  3.43  21.03  **69.53**  **3.86**  **0.43** |
| **HepB** | 0  1  2  **3**  **4**  **5** | 0.43  0.43  2.60  **96.54** | 2.58  19.07  **77.32**  **1.03** | 2.18  7.42  16.16  **67.69**  **6.55** | **100.0** | 0.41  9.02  **90.57** | 3.91  7.03  **80.86**  **7.81**  **0.39** | 0.40  2.37  9.88  **87.35** | 1.72  3.43  21.03  **69.53**  **3.86**  **0.43** |
| **HIB** | 0  1  2  **3**  **4**  **5** | 0.43  0.43  2.60  **96.54** | 1.55  5.15  14.43  **78.87** | 68.56  6.55  3.06  **20.09**  **1.75** | **100.0** | 1.23  **36.89**  **61.07**  **0.82** | 4.69  6.25  **89.06** | 0.40  3.16  8.30  **38.74**  **49.01**  **0.40** | 1.72  3.43  21.03  **69.53**  **3.86**  **0.43** |
| **Polio**  **OPV/IPV*** | 0  1  2  **3**  **4**  **5**  **6**  **7**  **8**  **9**  **10**  **11**  **12**  **13**  **14**  **15**  **16**  **17**  **18**  **19** | 0.43  0.43  **3.03**  **19.48**  **30.30**  **34.20**  **10.39**  **1.73** | 0.52  4.64  4.12  **15.98**  **21.13**  **28.87**  **11.34**  **11.34**  **2.06** | 1.75  **2.18**  **4.37**  **9.61**  **20.52**  **27.07**  **31.00**  **3.49** | **2.16**  **19.05**  **30.74**  **37.23**  **9.52**  **1.30** | 2.05  **75.82**  **3.28**  **18.85** | **0.39**  **0.78**  **1.95**  **4.30**  **12.11**  **19.92**  **21.88**  **17.97**  **11.72**  **6.64**  **1.56**  **0.78** | 0.79  1.19  2.77  9.49  **33.99**  **46.25**  **4.74**  **0.79** | 2.15  4.29  26.18  43.78  **20.60**  **2.15**  **0.86** |
| **Measles** | 0  **1**  **2**  **3** | 3.46  **71.86**  **23.38**  **1.30** | 26.80  **58.25**  **14.95** | 13.97  **30.13**  **53.71**  **2.18** | **17.75**  **62.77**  **19.48** | 9.84  **83.20**  **6.97** | 0.39  **19.14**  **69.92**  **10.55** | 10.67  **33.60**  **52.57**  **3.16** | 23.61  **72.53**  **3.86** |

**Supplementary Table 2**. Percent of children that have received a specific number of doses of a specific vaccine. The bold numbers show children fully covered; including birth doses but excluding scheduled booster doses. BGD: Dhaka, Bangladesh; BRF: Fortaleza, Brazil; INV: Vellore, India; NEB: Bhaktapur, Nepal; PEL: Loreto, Peru; PKN: Naushero Feroze, Pakistan; SAV: Venda, South Africa; TZH: Haydom, Tanzania. *IPV is only used in SAV.

|  | **Enrolled** | **Female/male** | **Lost to follow-up**  **(LTF)**  **(%)** | **Vaccinated according to BCG EPI schedule before LTF (%)** | **Vaccinated according to DPT EPI schedule before LTF (%)** | **Vaccinated according to Polio EPI schedule before LTF**  **(%)** |
| --- | --- | --- | --- | --- | --- | --- |
| **BGD** | 265 | 116/115 | 34 (13) | 30 (88) | 14 (41) | 13 (38) |
| **BRF** | 233 | 93/101 | 39 (17) | 31 (79) | 7 (18) | 11 (28) |
| **INV** | 251 | 124/105 | 22 (9) | 14 (64) | 7 (32) | 9 (41) |
| **NEB** | 240 | 108/123 | 9 (4) | 7 (78) | 7 (78) | 7 (78) |
| **PEL** | 303 | 110/134 | 59 (19) | 55 (93) | 30 (51) | 30 (51) |
| **PKN** | 277 | 130/126 | 21 (8) | 18 (86) | 11 (52) | 16 (76) |
| **SAV** | 314 | 126/127 | 61 (19) | 39 (64) | 18 (29) | 15 (25) |
| **TZH** | 262 | 116/117 | 29 (11) | 21 (72) | 8 (28) | 6 (21) |
| **Total** | 2145 | 923/948 | 274 (13) | 215 (78) | 102 (37) | 107 (39) |

**Supplementary Table 3**. Number of children enrolled, gender distribution, number of children lost to follow up, and number of children vaccinated according to EPI schedule prior to being lost to follow up. BGD: Dhaka, Bangladesh; BRF: Fortaleza, Brazil; INV: Vellore, India; NEB: Bhaktapur, Nepal; PEL: Loreto, Peru; PKN: Naushero Feroze, Pakistan; SAV: Venda, South Africa; TZH: Haydom, Tanzania.

|  |  | **Number of children**  **in household** | | | **Maternal age** | | | **Years of maternal education** | | |
| --- | --- | --- | --- | --- | --- | --- | --- | --- | --- | --- |
|  |  | **BCG1** | **DPT1** | **Measles1** | **BCG1** | **DPT1** | **Measles1** | **BCG1** | **DPT1** | **Measles1** |
| **BGD** | **Not on time**  **On time**  **p-value** | 1.93 (1.76-2.10)  1.89 (1.67-2.12)  0.81 | 2.05 (1.82-2.28)  1.85 (1.68-2.03)  0.18 | 1.94 (1.76-2.12)  1.89 (1.08-2.13)  0.75 | 24.9 (24.1-25.7)  25.1 (23.8-26.3)  0.80 | **25.8 (24.7-26.8)**  **24.5 (23.7-25.3)**  **0.08** | 24.8 (23.9-25.6)  25.2 (24.1-26.3)  0.52 | 4.5 (4.0-5.0)  4.9 (4.0-5.7)  0.47 | **4.1 (3.4-4.9)**  **4.9 (4.4-5.4)**  **0.09** | **4.3 (3.8-4.9)**  **5.1 (4.4-5.7)**  **0.09** |
| **BRF** | **Not on time**  **On time**  **p-value** | 2.44 (2.10-2.78)  2.27 (2.02-2.51)  0.40 | **2.62 (2.30-2.95)**  **2.08 (1.85-2.32)**  **<0.01** | 2.33 (2.10-2.55)  2.33 (1.88-2.78)  0.99 | 26.3 (25.1-27.6)  25.2 (24.2-26.2)  0.15 | 25.7 (24.5-26.8)  25.5 (24.5-26.6)  0.88 | 25.5 (24.6-26.3)  26.0 (24.0-28.0)  0.58 | 8.9 (8.3-9.5)  9.2 (8.6-9.7)  0.59 | 8.9 (8.4-9.5)  9.2 (8.6-9.8)  0.51 | 9.2 (8.8-9.6)  8.6 (7.5-9.7)  0.24 |
| **INV** | **Not on time**  **On time**  **p-value** | **2.45 (2.16-2.75)**  **1.88 (1.71-2.05)**  **<0.01** | **2.32 (2.08-2.56)**  **1.94 (1.71-2.17)**  **0.03** | **2.62 (2.20-3.04)**  **1.99 (1.82-2.16)**  **<0.01** | **25.0 (24.1-25.9)**  **23.8 (23.1-24.5)**  **0.04** | 24.6 (23.9-25.4)  24.1 (23.2-24.9)  0.34 | 25.1 (23.9-26.2)  24.1 (23.5-24.8)  0.14 | **6.2 (5.4-6.9)**  **7.6 (6.9-8.2)**  **<0.01** | **6.4 (5.7-7.1)**  **7.6 (6.9-8.3)**  **0.02** | 6.2 (5.2-7.3)  7.1 (6.6-7.7)  0.12 |
| **NEB** | **Not on time**  **On time**  **p-value** | 1.83 (1.61-2.04)  1.65 (1.54-1.76)  0.14 | 1.74 (1.55-1.93)  1.67 (1.55-1.79)  0.54 | 1.70 (1.59-1.82)  1.65 (1.45-1.85)  0.65 | 27.4 (26.4-28.4)  26.5 (26.0-27.1)  0.15 | 26.6 (25.6-27.6)  26.7 (26.2-27.3)  0.75 | 26.9 (26.3-27.4)  26.2 (25.2-27.2)  0.20 | **7.0 (5.8-8.1)**  **8.6 (8.0-9.1)**  **0.01** | **6.8 (5.6-7.9)**  **8.7 (8.1-9.2)**  **<0.01** | 8.3 (7.7-8.8)  8.0 (6.9-9.1)  0.72 |
| **PEL** | **Not on time**  **On time**  **p-value** | **3.14 (2.34-3.94)**  **2.34 (2.13-2.55)**  **0.01** | 2.37 (1.85-2.89)  2.44 (2.21-2.67)  0.81 | 2.49 (2.21-2.76)  2.35 (2.02-2.67)  0.52 | **26.5 (23.7-29.4)**  **24.4 (23.6-25.2)**  **0.08** | 24.9 (22.8-26.9)  24.6 (23.8-25.5)  0.85 | 24.9 (24.0-25.9)  24.3 (22.9-25.6)  0.42 | **6.5 (5.4-7.6)**  **7.9 (7.5-8.2)**  **0.01** | 7.4 (6.3-8.4)  7.7 (7.4-8.1)  0.43 | 7.9 (7.4-8.3)  7.4 (6.9-7.9)  0.18 |
| **PKN** | **Not on time**  **On time**  **p-value** | **3.56 (3.19-3.93)**  **2.83 (2.50-3.16)**  **<0.01** | 3.26 (2.96-3.56)  3.19 (2.68-3.69)  0.79 | 3.20 (2.90-3.50)  3.38 (2.87-3.89)  0.55 | 28.8 (27.9-29.8)  28.1 (27.0-29.2)  0.31 | 28.3 (27.5-29.1)  29.1 (27.4-30.7)  0.36 | 28.2 (27.3-29)  29.5 (28.1-31)  0.11 | 2.8 (2.2-3.5)  3.4 (2.6-4.2)  0.25 | 3.1 (2.5-3.7)  3.0 (1.9-4.1)  0.90 | 3.2 (2.6-3.8)  2.6 (1.7-3.6)  0.30 |
| **SAV** | **Not on time**  **On time**  **p-value** | 2.00 (0.93-3.07)  2.41 (2.19-2.62)  0.46 | **2.83 (2.34-3.31)**  **2.24 (2.01-2.47)**  **0.02** | 2.51 (2.23-2.78)  2.20 (1.87-2.53)  0.15 | 26.1 (20.5-31.8)  27.8 (26.7-28.9)  0.55 | **29.4 (27.1-31.8)**  **27.1 (26.0-28.3)**  **0.05** | 28.4 (27.0-29.8)  26.7 (25.1-28.2)  0.12 | 10.9 (10-11.8)  10.1 (9.8-10.3)  0.13 | 10.2 (9.6-10.7)  10.1 (9.8-10.4)  0.75 | **9.9 (9.6-10.3)**  **10.4 (10-10.7)**  **0.09** |
| **TZH** | **Not on time**  **On time**  **p-value** | 4.03 (3.69-4.36)  4.06 (3.10-5.02)  0.94 | 4.15 (3.78-4.51)  3.64 (3.03-4.25)  0.18 | 4.02 (3.69-4.35)  4.10 (3.15-5.05)  0.86 | 29.3 (28.4-30.2)  30.3 (27.9-32.7)  0.43 | 29.7 (28.7-30.7)  28.7 (27.0-30.3)  0.29 | 29.5 (28.6-30.4)  29.5 (27.0-32.1)  0.95 | 5.1 (4.7-5.5)  5.1 (4.2-6.0)  0.97 | 5.0 (4.6-5.4)  5.2 (4.4-6.0)  0.73 | 5.1 (4.7-5.5)  4.8 (3.7-5.8)  0.52 |

**Supplementary Table 4**. Socioeconomic characteristics association with compliance with country specific EPI schedule analyzed using Students t-test (95% confidence intervals). Adherence is defined as vaccine administration within 7 days of site specific EPI schedule. BCG1: first dose of BCG; DPT1: first dose of DPT; Measles1: first dose of measles; BGD: Dhaka, Bangladesh; BRF: Fortaleza, Brazil; INV: Vellore, India; NEB: Bhaktapur, Nepal; PEL: Loreto, Peru; PKN: Naushero Feroze, Pakistan; SAV: Venda, South Africa; TZH: Haydom, Tanzania. Bold numbers indicate a significant finding: p-value<0.10

|  |  | **WAMI** | | | **Household income**  **(US dollars)** | | |
| --- | --- | --- | --- | --- | --- | --- | --- |
|  |  | **BCG1** | **DPT1** | **Measles1** | **BCG1** | **DPT1** | **Measles1** |
| **BGD** | **Not on time**  **On time**  **p-value** | 0.52 (0.50-0.54)  0.55 (0.52-0.59)  0.12 | 0.52 (0.49-0.55)  0.53 (0.51-0.55)  0.40 | 0.53 (0.50-0.55)  0.53 (0.51-0.56)  0.63 | 126 (108-143)  132 (112-152)  0.69 | 127 (107-146)  128 (109-146)  0.93 | 131 (113-148)  122 (99-145)  0.57 |
| **BRF** | **Not on time**  **On time**  **p-value** | 0.82 (0.80-0.85)  0.82 (0.80-0.84)  0.91 | 0.81 (0.79-0.83)  0.83 (0.81-0.85)  0.27 | 0.82 (0.81-0.84)  0.81 (0.77-0.85)  0.48 | 348 (316-381)  356 (329-383)  0.71 | 341 (311-371)  363 (335-392)  0.28 | 356 (334-379)  340 (286-394)  0.53 |
| **INV** | **Not on time**  **On time**  **p-value** | **0.42 (0.39-0.45)**  **0.48 (0.45-0.50)**  **<0.01** | **0.43 (0.41-0.46)**  **0.47 (0.45-0.50)**  **0.04** | **0.41 (0.37-0.45)**  **0.47 (0.44-0.49)**  **<0.01** | 73 (64-82)  78 (69-87)  0.51 | 74 (66-83)  77 (67-87)  0.70 | 78 (64-93)  75 (67-82)  0.60 |
| **NEB** | **Not on time**  **On time**  **p-value** | **0.67 (0.62-0.71)**  **0.71 (0.69-0.73)**  **0.04** | 0.68 (0.65-0.71)  0.71 (0.69-0.73)  0.20 | 0.70 (0.68-0.72)  0.70 (0.67-0.73)  0.93 | 192 (148-237)  190 (169-210)  0.91 | 172 (147-197)  196 (173-219)  0.27 | 188 (167-2090  196 (157-234)  0.72 |
| **PEL** | **Not on time**  **On time**  **p-value** | **0.50 (0.46-0.54)**  **0.54 (0.53-0.56)**  **0.08** | 0.54 (0.49-0.59)  0.54 (0.52-0.56)  0.89 | 0.54 (0.52-0.56)  0.54 (0.51-0.56)  0.90 | 122 (100-144)  140 (130-150)  0.22 | 149 (117-181)  136 (126-145)  0.31 | 143 (129-156)  130 (119-142)  0.21 |
| **PKN** | **Not on time**  **On time**  **p-value** | 0.48 (0.45-0.51)  0.50 (0.47-0.54)  0.31 | **0.50 (0.47-0.53)**  **0.45 (0.41-0.49)**  **0.05** | 0.49 (0.46-0.52)  0.48 (0.43-0.53)  0.63 | 183 (159-207)  167 (144-190)  0.36 | **193 (170-215)**  **132 (115-149)**  **<0.01** | 175 (167-194)  178 (138-217)  0.90 |
| **SAV** | **Not on time**  **On time**  **p-value** | 0.81 (0.69-0.92)  0.75 (0.73-0.77)  0.26 | 0.75 (0.70-0.80)  0.75 (0.73-0.77)  0.84 | 0.75 (0.72-0.77)  0.76 (0.73-0.79)  0.56 | 218 (108-328)  253 (219-287)  0.69 | **314 (224-404)**  **230 (198-261)**  **0.03** | 256 (216-296)  244 (186-303)  0.73 |
| **TZH** | **Not on time**  **On time**  **p-value** | 0.21 (0.20-0.23)  0.19 (0.15-0.23)  0.32 | 0.21 (0.19-0.23)  0.20 (0.17-0.24)  0.69 | 0.21 (0.20-0.23)  0.19 (0.15-0.24)  0.38 | 29 (23-24)  29 (16-43)  0.90 | 30 (24-36)  24 (18-31)  0.32 | 28 (23-33)  31 (13-50)  0.66 |

**Supplementary Table 4 continued.**

|  | **Sex** |  |  |  |  |
| --- | --- | --- | --- | --- | --- |
|  |  | **BCG1**  **On time** | **DPT1**  **On time** | **Measles1**  **On time** | **Fully vaccinated** |
| **BGD** | **Female**  **Male**  **P-value** | **0.31 (0.23-0.39)**  **0.19 (0.12-0.26)**  **0.04** | 0.69 (0.60-0.77)  0.63 (0.54-0.71)  0.31 | 0.39 (0.30-0.48)  0.39 (0.30-0.48)  0.96 | 0.93 (0.88-0.98)  0.95 (0.91-0.99)  0.59 |
| **BRF** | **Female**  **Male**  **P-value** | 0.69 (0.59-0.78)  0.59 (0.50-0.69)  0.17 | 0.54 (0.44-0.64)  0.55 (0.46-0.65)  0.81 | 0.18 (0.10-0.26)  0.22 (0.14-0.30)  0.54 | **0.53 (0.42-0.63)**  **0.65 (0.56-0.75)**  **0.07** |
| **INV** | **Female**  **Male**  **P-value** | 0.53 (0.44-0.62)  0.51 (0.42-0.61)  0.79 | 0.42 (0.33-0.51)  0.46 (0.36-0.55)  0.57 | 0.70 (0.62-0.78)  0.78 (0.70-0.86)  0.17 | 0.74 (0.66-0.82)  0.83 (0.76-0.90­)  0.11 |
| **NEB*** | **Female**  **Male**  **P-value** | 0.81 (0.74-0.89)  0.74 (0.66-0.82)  0.17 | 0.78 (0.70-0.86)  0.72 (0.64-0.80)  0.34 | 0.29 (0.20-0.37)  0.26 (0.18-0.34)  0.65 | - |
| **PEL** | **Female**  **Male**  **P-value** | 0.88 (0.82-0.94)  0.88 (0.83-0.93)  0.98 | 0.83 (0.76-0.90)  0.87 (0.82-0.93)  0.31 | 0.41 (0.32-0.50)  0.37 (0.29-0.45)  0.57 | 0.86 (0.80-0.93)  0.86 (0.80-0.92)  0.90 |
| **PKN** | **Female**  **Male**  **P-value** | 0.43 (0.35-0.52)  0.44 (0.36-0.53)  0.82 | 0.26 (0.19-0.34)  0.29 (0.21-0.36)  0.66 | **0.31 (0.23-0.39)**  **0.17 (0.11-0.24)**  **<0.01** | 0.91 (0.86-0.96)  0.86 (0.80-0.92)  0.21 |
| **SAV** | **Female**  **Male**  **P-value** | 0.95 (0.91-0.99)  0.93 (0.88-0.97)  0.43 | 0.67 (0.58-0.75)  0.75 (0.67-0.82)  0.15 | 0.35 (0.27-0.43)  0.40 (0.32-0.49)  0.39 | 0.74 (0.66-0.81)  0.72 (0.64-0.79)  0.70 |
| **TZH** | **Female**  **Male**  **P-value** | 0.14 (0.07-0.20)  0.14 (0.08-0.21)  0.87 | 0.24 (0.16-0.32)  0.21 (0.14-0.29)  0.61 | 0.15 (0.09-0.22)  0.09 (0.04-0.15)  0.16 | 0.17 (0.10-0.24)  0.14 (0.07-0.20)  0.45 |

**Supplementary Table 5**. Vaccination schedule compliance status by gender and whether or not the child is first born analyzed by comparison of the proportions (95% confidence intervals) of children vaccinated on time and fully or not fully vaccinated children. Adherence is defined as vaccine administration within 7 days of site specific EPI schedule. BCG1: first dose of BCG; DPT1: first dose of DPT; Measles1: first dose of measles; BGD: Dhaka, Bangladesh; BRF: Fortaleza, Brazil; INV: Vellore, India; NEB: Bhaktapur, Nepal; PEL: Loreto, Peru; PKN: Naushero Feroze, Pakistan; SAV: Venda, South Africa; TZH: Haydom, Tanzania. *All children in NEB were fully vaccinated. Bold numbers indicate a significant finding: p-value<0.10

|  | **First child** |  |  |  |  |
| --- | --- | --- | --- | --- | --- |
|  |  | **BCG1**  **On time** | **DPT1**  **On time** | **Measles1**  **On time** | **Fully Vaccinated** |
| **BGD** | **Not 1st child**  **1st child**  **P-value** | 0.29 (0.21-0.36)  0.21 (0.12-0.30)  0.21 | **0.61 (0.53-0.70)**  **0.73 (0.63-0.82)**  **0.08** | 0.38 (0.30-0.46)  0.40 (0.30-0.50)  0.75 | 0.94 (0.90-0.98)  0.94 (0.89-0.99)  0.96 |
| **BRF** | **Not 1st child**  **1st child**  **P-value** | **0.60 (0.51-0.68)**  **0.73 (0.61-0.84)**  **0.08** | **0.48 (0.40-0.57)**  **0.68 (0.56-0.79)**  **0.01** | 0.20 (0.13-0.26)  0.21 (0.11-0.31)  0.84 | 0.58 (0.50-0.67)  0.61 (0.49-0.73)  0.70 |
| **INV** | **Not 1st child**  **1st child**  **P-value** | 0.50 (0.42-0.58)  0.59 (0.47-0.70)  0.20 | **0.40 (0.32-0.47)**  **0.52 (0.41-0.63)**  **0.08** | **0.69 (0.61-0.76)**  **0.84 (0.76-0.92)**  **0.01** | **0.71 (0.64-0.79)**  **0.91 (0.84-0.97)**  **<0.01** |
| **NEB*** | **Not 1st child**  **1st child**  **P-value** | **0.72 (0.65-0.80)**  **0.84 (0.77-0.91)**  **0.04** | 0.73 (0.66-0.81)  0.77 (0.69-0.85)  0.52 | 0.24 (0.17-0.32)  0.31 (0.22-0.40)  0.27 | - |
| **PEL** | **Not 1st child**  **1st child**  **P-value** | 0.87 (0.81-0.92)  0.90 (0.84-0.96)  0.40 | 0.83 (0.77-0.89)  0.88 (0.82-0.95)  0.31 | 0.37 (0.29-0.45)  0.42 (0.32-0.52)  0.45 | 0.85 (0.0.8-0.91)  0.87 (0.80-0.94)  0.71 |
| **PKN** | **Not 1st child**  **1st child**  **P-value** | **0.41 (0.34-0.47)**  **0.54 (0.40-0.67)**  **0.09** | 0.27 (0.21-0.33)  0.29 (0.17-0.40)  0.83 | 0.27 (0.20-0.33)  0.18 (0.08-0.28)  0.18 | 0.88 (0.83-0.92)  0.89 (0.81-0.97)  0.78 |
| **SAV** | **Not 1st child**  **1st child**  **P-value** | 0.97 (0.93-1)  0.95 (0.89-1)  0.59 | 0.72 (0.64-0.80)  0.78 (0.68-0.89)  0.34 | 0.35 (0.26-0.43)  0.47 (0.34-0.59)  0.13 | 0.71 (0.63-0.79)  0.70 (0.58-0.82)  0.91 |
| **TZH** | **Not 1st child**  **1st child**  **P-value** | 0.14 (0.09-0.19)  0.18 (0.04-0.33)  0.52 | 0.23 (0.17-0.28)  0.26 (0.09-0.42)  0.71 | 0.13 (0.08-0.17)  0.11 (-0.01-0.23)  0.80 | 0.15 (0.10-0.20)  0.22 (0.06-0.38)  0.32 |

**Supplementary Table 5 continued.**

|  |  | **BCG**  **1st dose** | **DPT**  **3rd dose** | **OPV**  **3rd dose** | **Measles**  **1st dose** |
| --- | --- | --- | --- | --- | --- |
| **Bangladesh** | **MAL-ED study cohort**  DHS (2011)  WHO/UNICEF | **100**  97  99 | **96**  93  97 | **99**  93  97 | **97**  90  93 |
| **Brazil** | **MAL-ED study cohort**  DHS (1996) WHO/UNICEF | **100**  93  99 | **80**  84  95 | **91**  85  99 | **73**  91  99 |
| **India** | **MAL-ED study cohort**  DHS (2005-2006)  WHO/UNICEF | **99**  78  87 | **89**  60  72 | **100**  80  70 | **86**  63  74 |
| **Nepal** | **MAL-ED study cohort**  DHS (2011)  WHO/UNICEF | **100**  94  97 | **100**  92  92 | **100**  92  92 | **100**  90  88 |
| **Peru** | **MAL-ED study cohort**  DHS (2012)  WHO/UNICEF | **99**  92  95 | **98**  78  88 | **98**  84  71 | **90**  87  85 |
| **Pakistan** | **MAL-ED study cohort**  DHS (2012-2013)  WHO/UNICEF | **98**  80  85 | **89**  61  72 | **100**  81  72 | **100***  63  61 |
| **South Africa** | **MAL-ED study cohort**  DHS (1998)  WHO/UNICEF | **97**  94  84 | **86**  72  65 | **10****  65  66 | **89**  85  66 |
| **Tanzania** | **MAL-ED study cohort**  DHS (2010)  WHO/UNICEF | **87**  96  99 | **74**  90  91 | **67**  85  91 | **76**  88  99 |

**Supplementary Table 6**. Comparison of MAL-ED data with DHS, WHO data. DHS numbers are obtained from the most recent DHS available with the year shown in parentheses. Children who are vaccinated includes vaccinations recorded on the vaccine card, reported by the mother, and vaccines reported on the card but no date available. The children were age 1 to 4 years and alive at the time of questionnaire administration. WHO/UNICEF numbers are national estimates from 2013 . *One child did not receive the measles vaccine. **In SAV there are only 2 doses of OPV on the EPI schedule which explains why very few receive a 3rd dose of the vaccine.

**Supplementary Figure 1.** Total number of polio doses (OPV and/or IPV) received by children at 24 months of age. BGD: Dhaka, Bangladesh; BRF: Fortaleza, Brazil; INV: Vellore, India; NEB: Bhaktapur, Nepal; PEL: Loreto, Peru; PKN: Naushero Feroze, Pakistan; SAV: Venda, South Africa; TZH: Haydom, Tanzania.
