# Supplementary material for: Vaccine coverage and adherence to EPI schedules in eight resource poor settings in the MAL-ED cohort study
Source: Vaccine. 2017 Jan 11;35(3):443–51. doi: 10.1016/j.vaccine.2016.11.075 (PMC5244255; doi:10.1016/j.vaccine.2016.11.075)
Supplement: Supplementary data 2 — MAL-ED Network Investigators – complete list. [file mmc2.docx]

**MAL-ED Network Investigators**

**Writing group:** Christel Hoest, Jessica C. Seidman, Gwenyth Lee, James A Platts-Mills, Asad Ali, Maribel Paredes Olortegui, Pascal Bessong, Ram Chandyo, Sudhir Babji, Venkata Raghava Mohan, Dinesh Mondal, Mustafa Mahfuz, MBBS, Estomih R Mduma, Emanuel Nyathi, Claudia Abreu, Mark Miller, Carl J. Mason, and Stacey Knobler

**Data analysis:** Christel Hoest and Jessica C. Seidman

**Project management:**

Rebecca R Blank^7^, [rebecca.blank@gmail.com](mailto:rebecca.blank@gmail.com)

Michael Gottlieb^7^, [mgottlieb@fnih.org](mailto:mgottlieb@fnih.org)

Stacey L Knobler^6^, [Stacey.Knobler@nih.gov](mailto:Stacey.Knobler@nih.gov)

Dennis R Lang^6, 7^, [Lang4@fnih.org](mailto:Lang4@fnih.org)

Mark A Miller^6^, [Mark.Miller3@nih.gov](mailto:Mark.Miller3@nih.gov)

Karen H Tountas^7^, [ktountas@fnih.org](mailto:ktountas@fnih.org)

**Project technical subcommittee leadership:**

Zulfiqar A Bhutta^2^, [zulfiqar.bhutta@aku.edu](mailto:zulfiqar.bhutta@aku.edu)

Laura Caulfield^11^, [lcaulfi1@jhu.edu](mailto:lcaulfi1@jhu.edu)

William Checkley^11, 6^, [wcheckl1@jhmi.edu](mailto:wcheckl1@jhmi.edu)

Richard L Guerrant^18^, [guerrant@virginia.edu](mailto:guerrant@virginia.edu)

Eric Houpt^18^, [erh6k@virginia.edu](mailto:erh6k@virginia.edu)

Margaret N Kosek^11^, [mkosek@jhmi.edu](mailto:mkosek@jhmi.edu)

Dennis R Lang^6, 7^, [Lang4@FNIH.org](mailto:Lang4@FNIH.org)

Carl J Mason^3^, [carlmason@icloud.com](mailto:carlmason@icloud.com)

Mark A Miller^6^, [Mark.Miller3@nih.gov](mailto:Mark.Miller3@nih.gov)

Laura E Murray-Kolb^12^, [lem118@psu.edu](mailto:lem118@psu.edu)

William A Petri, Jr. ^18^, [wap3g@virginia.edu](mailto:wap3g@virginia.edu)

Jessica C Seidman^6^, [Jessica.Seidman@nih.gov](mailto:Jessica.Seidman@nih.gov)

**Study site lead investigators:**

Tahmeed Ahmed^9^, [tahmeed@icddrb.org](mailto:tahmeed@icddrb.org)

Pascal Bessong^17^, [pascal.bessong@univen.ac.za](mailto:pascal.bessong@univen.ac.za)

Zulfiqar A Bhutta^2^, [zulfiqar.bhutta@aku.edu](mailto:zulfiqar.bhutta@aku.edu)

Rashidul Haque^9^, [rhaque@icddrb.org](mailto:rhaque@icddrb.org)

Sushil John^4^, [rikkisush@cmcvellore.ac.in](mailto:rikkisush@cmcvellore.ac.in)

Gagandeep Kang^4^, [gkang@cmcvellore.ac.in](mailto:gkang@cmcvellore.ac.in)

Margaret N Kosek^11^, [mkosek@jhmi.edu](mailto:mkosek@jhmi.edu)

Aldo AM Lima^14^, [alima@ufc.br](mailto:alima@ufc.br)

Estomih R Mduma^8^, [estomih.mduma@haydom.co.tz](mailto:estomih.mduma@haydom.co.tz)

Reinaldo B Oriá^14^, [rbo5u@hscmail.mcc.virginia.edu](mailto:rbo5u@hscmail.mcc.virginia.edu)

Prakash Sunder Shrestha^10^, [prakashsunder@hotmail.com](mailto:prakashsunder@hotmail.com)

Sanjaya Kumar Shrestha^19^, [ShresthaSK@afrims.org](mailto:ShresthaSK@afrims.org)

Erling Svensen^20, 8^, [Erling.Svensen@cih.uib.no](mailto:Erling.Svensen@cih.uib.no)

Anita KM Zaidi^2^, [anita.zaidi@aku.edu](mailto:anita.zaidi@aku.edu)

**Data and sample collection and management:**

Cláudia B Abreu^14^, [claudia_beghini2004@yahoo.com.br](mailto:claudia_beghini2004@yahoo.com.br)

Angel Mendez Acosta^1^, [amendez@prisma.org.pe](mailto:amendez@prisma.org.pe)

Imran Ahmed^2^, [imran.ahmed@aku.edu](mailto:imran.ahmed@aku.edu)

AM Shamsir Ahmed^9^, [a.ahmed@uq.net.au](file:///C:\Users\Dennis\Downloads\a.ahmed@uq.net.au)

Asad Ali^2^, [asad.ali@aku.edu](mailto:asad.ali@aku.edu)

Ramya Ambikapathi^6^, [rambikapathi@gmail.com](mailto:rambikapathi@gmail.com)

Leah Barrett^18^, [ljbarrett@mindspring.com](https://mail.nih.gov/owa/redir.aspx?SURL=ylNCiUwlIdiB8hZ2YD5o5PjURw5tc7rmQlR7x314W-eQKQJUbwLTCG0AYQBpAGwAdABvADoAbABqAGIAYQByAHIAZQB0AHQAQABtAGkAbgBkAHMAcAByAGkAbgBnAC4AYwBvAG0A&URL=mailto%3aljbarrett%40mindspring.com)

Aubrey Bauck^11^, [abauck1@jhu.edu](mailto:abauck1@jhu.edu)

Eliwaza Bayo^8^, [elb.bayo@gmail.com](https://kalender.uib.no/owa/redir.aspx?SURL=ub_2qWHkBTDGrgIHTjqmsjQyS_vF_c9EfXjHVufZnQVUeflVyALTCG0AYQBpAGwAdABvADoAZQBsAGIALgBiAGEAeQBvAEAAZwBtAGEAaQBsAC4AYwBvAG0A&URL=mailto%3aelb.bayo%40gmail.com)

Ladaporn Bodhidatta^3^, [ladapornb@afrims.org](mailto:ladapornb@afrims.org)

Anuradha Bose^4^, [abose@cmcvellore.ac.in](mailto:abose@cmcvellore.ac.in)

J Daniel Carreon^6^, [carreonj@mail.nih.gov](mailto:carreonj@mail.nih.gov)

Ram Krishna Chandyo^10^, [ram.chandyo@uib.no](mailto:ram.chandyo@uib.no)

Vivek Charu^6^, [vcharu@jhsph.edu](mailto:vcharu@jhsph.edu)

Hilda Costa^14^, [hildacosta@hotmail.com](mailto:hildacosta@hotmail.com)

Leyfou Dabo^6^, [lfdabo@gmail.com](mailto:lfdabo@gmail.com)

Rebecca Dillingham^18^, [rd8v@virginia.edu](https://mail.nih.gov/owa/redir.aspx?SURL=JtHDEfZTn_iErPWJ-iQHJx2-1fDUbCv5MkGVAUrfHviQKQJUbwLTCG0AYQBpAGwAdABvADoAcgBkADgAdgBAAHYAaQByAGcAaQBuAGkAYQAuAGUAZAB1AA..&URL=mailto%3ard8v%40virginia.edu)

Alessandra Di Moura^14^, [ferrer.alessandra@yahoo.com.br](file:///C:\Users\doanv\Documents\MAL-ED\E-mail%20List\ferrer.alessandra@yahoo.com.br)

Viyada Doan^6^, [Viyada.Doan@nih.gov](mailto:Viyada.Doan@nih.gov)

Jose Quirino Filho^14, 6^, [jqf_ce@yahoo.com.br](mailto:jqf_ce@yahoo.com.br)

Jhanelle Graham^6^, [jhalexia@gmail.com](file:///C:\Users\doanv\Documents\MAL-ED\E-mail%20List\jhalexia@gmail.com)

Christel Hoest^6^, [christel.host@nih.gov](mailto:christel.host@nih.gov)

Iqbal Hossain^9^, [ihossain@icddrb.org](mailto:ihossain@icddrb.org)

Munirul Islam^9^, [mislam@icddrb.org](https://mail.nih.gov/owa/redir.aspx?SURL=koV1njEhW_TBe7SX1sujsl8Hy3EfA_bf4SB9Ncicl133kTgFhwXTCG0AYQBpAGwAdABvADoAbQBpAHMAbABhAG0AQABpAGMAZABkAHIAYgAuAG8AcgBnAA..&URL=mailto%3amislam%40icddrb.org)

M Steffi Jennifer^4^, [stefjeni.11@gmail.com](file:///C:\Users\doanv\Documents\MAL-ED\E-mail%20List\stefjeni.11@gmail.com)

Shiny Kaki^4^, [shinykaki@gmail.com](file:///C:\Users\doanv\Documents\MAL-ED\E-mail%20List\shinykaki@gmail.com)

Beena Koshy^4^, [beenakoshy1@rediffmail.com](mailto:beenakoshy1@rediffmail.com)

Gwenyth Lee^11^, [gwenyth.lee@gmail.com](mailto:gwenyth.lee@gmail.com)

Álvaro M Leite^14^, [alvaromadeiro@yahoo.com.br](file:///C:\Users\doanv\Documents\MAL-ED\E-mail%20List\alvaromadeiro@yahoo.com.br)

Noélia L Lima^14^, [noelialima30@yahoo.com.br](file:///C:\Users\doanv\Documents\MAL-ED\E-mail%20List\noelialima30@yahoo.com.br)

Bruna LL Maciel^14^, [brunalimamaciel@gmail.com](file:///C:\Users\doanv\Documents\MAL-ED\E-mail%20List\brunalimamaciel@gmail.com)

Mustafa Mahfuz^9^, [mustafa@icddrb.org](mailto:mustafa@icddrb.org)

Cloupas Mahopo^17^, [mahopotc@gmail.com](mailto:mahopotc@gmail.com)

Angelina Maphula^17^, [angelina.maphula@univen.ac.za](mailto:angelina.maphula@univen.ac.za)

Benjamin JJ McCormick^6^, [ben.mccormick@gmail.com](mailto:ben.mccormick@gmail.com)

Monica McGrath^6^, [mcgrath.monica@gmail.com](mailto:mcgrath.monica@gmail.com)

Archana Mohale^6^, [mohalea@mail.nih.gov](mailto:mohalea@mail.nih.gov)

Milena Moraes^14^, [milenamaia@hotmail.com](mailto:milenamaia@hotmail.com)

Francisco S Mota^14^, [sulivan.mota@iprede.org.br](mailto:sulivan.mota@iprede.org.br)

Jayaprakash Muliyil^4^, [jpmuliyil@gmail.com](file:///C:\Users\doanv\Documents\MAL-ED\E-mail%20List\jpmuliyil@gmail.com)

Regisiana Mvungi^8^, [regisiana@yahoo.com](file:///C:\Users\doanv\Documents\MAL-ED\E-mail%20List\regisiana@yahoo.com)

Gaurvika Nayyar^6^, [gaurvika@gmail.com](file:///C:\Users\doanv\Documents\MAL-ED\E-mail%20List\gaurvika@gmail.com)

Emanuel Nyathi^17^, [Emanuel.Nyathi@univen.ac.za](mailto:Emanuel.Nyathi@univen.ac.za)

Maribel Paredes Olortegui^1^, [mparedeso@prisma.org.pe](mailto:mparedeso@prisma.org.pe)

Reinaldo Oria^14^, [rbo5u@hscmail.mcc.virginia.edu](mailto:rbo5u@hscmail.mcc.virginia.edu)

Angel Orbe Vasquez^1^, [angel_orbe@hotmail.com](mailto:angel_orbe@hotmail.com)

William K Pan^5, 6^, [william.pan@duke.edu](mailto:william.pan@duke.edu)

John Pascal^8^, [johnagustinopaschal@gmail.com](https://kalender.uib.no/owa/redir.aspx?SURL=1qXGbGQk4CjeysFkypEyZpchGc-JZDOguiT2jn_SOs9UeflVyALTCG0AYQBpAGwAdABvADoAagBvAGgAbgBhAGcAdQBzAHQAaQBuAG8AcABhAHMAYwBoAGEAbABAAGcAbQBhAGkAbAAuAGMAbwBtAA..&URL=mailto%3ajohnagustinopaschal%40gmail.com)

Crystal L Patil^16^, [cpatil@uic.edu](mailto:cpatil@uic.edu)

Laura Pendergast^13^, [laura.pendergast@temple.edu](mailto:laura.pendergast@temple.edu)

Silvia Rengifo Pinedo^1^, [siguase36@hotmail.com](file:///C:\Users\Dennis\Downloads\siguase36@hotmail.com)

James Platts-Mills^18^, [jp5t@hscmail.mcc.virginia.edu](mailto:jp5t@hscmail.mcc.virginia.edu)

Stephanie Psaki^6^, [spsaki@popcouncil.org](file:///C:\Users\doanv\Documents\MAL-ED\E-mail%20List\spsaki@popcouncil.org)

Mohan Venkata Raghava^4^, [venkat@cmcvellore.ac.in](mailto:venkat@cmcvellore.ac.in)

Karthikeyan Ramanujam^4^, [karthikeyan05@yahoo.co.in](mailto:karthikeyan05@yahoo.co.in)

Muneera Rasheed^2^, [muneera.rasheed@aku.edu](mailto:muneera.rasheed@aku.edu)

Zeba A Rasmussen^6^, [Zeba.Rasmussen@nih.gov](mailto:Zeba.Rasmussen@nih.gov)

Stephanie A Richard^6^, [Stephanie.Richard@nih.gov](mailto:Stephanie.Richard@nih.gov)

Anuradha Rose^4^, [anurose@cmcvellore.ac.in](file:///C:\Users\doanv\Documents\MAL-ED\E-mail%20List\anurose@cmcvellore.ac.in)

Reeba Roshan^4^, [reebageorge@hotmail.com](mailto:reebageorge@hotmail.com)

Barbara Schaefer^12, 6^, [bas19@psu.edu](mailto:bas19@psu.edu)

Rebecca Scharf^18^, [rebeccascharf@virginia.edu](mailto:rebeccascharf@virginia.edu)

Jessica C Seidman^6^, [Jessica.Seidman@nih.gov](mailto:Jessica.Seidman@nih.gov)

Srujan L Sharma^4^, [srujan.sharma@gmail.com](mailto:srujan.sharma@gmail.com)

Binob Shrestha^19^, [binobs@afrims.org](mailto:binobs@afrims.org)

Rita Shrestha^10^, [ritas_12@yahoo.com](mailto:ritas_12@yahoo.com)

Suzanne Simons^12^, [sxs126@psu.edu](mailto:sxs126@psu.edu)

Alberto M Soares ^14^, [soaresam@ufc.br](file:///C:\Users\doanv\Documents\MAL-ED\E-mail%20List\soaresam@ufc.br)

Rosa MS Mota ^14^, [rosa@dema.ufc.br](file:///C:\Users\doanv\Documents\MAL-ED\E-mail%20List\rosa@dema.ufc.br)

Sajid Soofi^2^, [sajid.soofi@aku.edu](mailto:sajid.soofi@aku.edu)

Tor Strand^19, 15^, [Tors@me.com](file:///C:\Users\doanv\Documents\MAL-ED\E-mail%20List\Tors@me.com)

Fahmida Tofail^9^, [ftofail@icddrb.org](mailto:ftofail@icddrb.org)

Rahul J Thomas^4^, [rj_thomas99@yahoo.com](file:///C:\Users\doanv\Documents\MAL-ED\E-mail%20List\rj_thomas99@yahoo.com)

Ali Turab^2^, [turab.ali@aku.edu](mailto:turab.ali@aku.edu)

Manjeswori Ulak^10^, [manjeswori@gmail.com](mailto:manjeswori@gmail.com)

Vivian Wang^6^, [aijun.wang@nih.gov](mailto:aijun.wang@nih.gov)

Ladislaus Yarrot^8^, [ladisblacy@yahoo.com](mailto:ladisblacy@yahoo.com)

Pablo Peñataro Yori^11^, [pyori@jhsph.edu](mailto:pyori@jhsph.edu)

Aisha K Yousafzai^2^, [aisha.yousafzai@aku.edu](mailto:aisha.yousafzai@aku.edu)

**Sample processing and management:**

Didar Alam^2^, [didar.alam@aku.edu](mailto:didar.alam@aku.edu)

Ramya Ambikapathi^6^, [rambikapathi@gmail.com](mailto:rambikapathi@gmail.com)

Caroline Amour^8^, [lyneamour@gmail.com](mailto:lyneamour@gmail.com)

Cesar Banda Chavez^1^, [cebchavez@yahoo.com](mailto:cebchavez@yahoo.com)

Sudhir Babji^4^, [sudhirbabji@cmcvellore.ac.in](mailto:sudhirbabji@cmcvellore.ac.in)

Viyada Doan^6^, [Viyada.Doan@nih.gov](mailto:Viyada.Doan@nih.gov)

Julian Torres Flores^1^, [jflores@prisma.org.pe](file:///C:\Users\doanv\Documents\MAL-ED\E-mail%20List\jflores@prisma.org.pe)

Jean Gratz^18^, [jean.gratz@gmail.com](mailto:jean.gratz@gmail.com)

Alexandre Havt^14^, [ahavtbinda@gmail.com](mailto:ahavtbinda@gmail.com)

Eric Houpt^18^, [erh6k@virginia.edu](mailto:erh6k@virginia.edu)

Ila F Lima^14^, [ilafarm@yahoo.com.br](mailto:ilafarm@yahoo.com.br)

Monica McGrath^6^, [mcgrath.monica@gmail.com](mailto:mcgrath.monica@gmail.com)

Dinesh Mondal^9^, [din63d@icddrb.org](mailto:din63d@icddrb.org)

Rosemary Nshama^8^, [nshamarosemary@yahoo.com](https://kalender.uib.no/owa/redir.aspx?SURL=44eKywibT_VxkVYbqwNq_iu9dnbRDY3HdobxvhwMxEdUeflVyALTCG0AYQBpAGwAdABvADoAbgBzAGgAYQBtAGEAcgBvAHMAZQBtAGEAcgB5AEAAeQBhAGgAbwBvAC4AYwBvAG0A&URL=mailto%3anshamarosemary%40yahoo.com)

Pedro HQS Medeiros^14^, [phquintela@hotmail.com](file:///C:\Users\doanv\Documents\MAL-ED\E-mail%20List\phquintela@hotmail.com)

Shahida Qureshi^2^, [shahida.qureshi@aku.edu](mailto:shahida.qureshi@aku.edu)

Anup Ramachandran^4^, [anuprama@yahoo.co.uk](file:///C:\Users\doanv\Documents\MAL-ED\E-mail%20List\anuprama@yahoo.co.uk)

A Catharine Ross^12^, [Acr6@psu.edu](https://mail.nih.gov/owa/redir.aspx?SURL=T_xGdajVnTwLywZ9Elh5LdwZz8FeOA4CJRlxVLMvT8SwxvYObALTCG0AYQBpAGwAdABvADoAQQBjAHIANgBAAHAAcwB1AC4AZQBkAHUA&URL=mailto%3aAcr6%40psu.edu)

Amidou Samie^17^, [samieamidou@yahoo.com](mailto:samieamidou@yahoo.com)

Kerry Schulze^11^, [kschulz1@jhu.edu](mailto:kschulz1@jhu.edu)

Jessica C Seidman^6^, [Jessica.Seidman@nih.gov](mailto:Jessica.Seidman@nih.gov)

Buliga Mujaga Swema^8^, [buligamujaga@yahoo.co.uk](https://kalender.uib.no/owa/redir.aspx?SURL=cnWumhcJ3YsHsQLZv_XfcEknGOYFFLkGvO1QIRmMpkZUeflVyALTCG0AYQBpAGwAdABvADoAYgB1AGwAaQBnAGEAbQB1AGoAYQBnAGEAQAB5AGEAaABvAG8ALgBjAG8ALgB1AGsA&URL=mailto%3abuligamujaga%40yahoo.co.uk)

Mery Siguas Salas^1^, [msiguas@prisma.org.pe](file:///C:\Users\doanv\Documents\MAL-ED\E-mail%20List\msiguas@prisma.org.pe)

Dixner Rengifo Trigoso^1^, [drengifo@prisma.org.pe](file:///C:\Users\doanv\Documents\MAL-ED\E-mail%20List\drengifo@prisma.org.pe)

Rosa Rios de Burga^1^, [rosaburga@gmail.com](file:///C:\Users\doanv\Documents\MAL-ED\E-mail%20List\rosaburga@gmail.com)

**Affiliations** *(these will be matched w/ numbers to each investigators once the investigator list is finalized)*

^1^A.B. PRISMA, Iquitos, Peru

^2^Aga Khan University, Karachi, Pakistan

^3^Armed Forces Research Institute of Medical Sciences, Bangkok, Thailand

^4^Christian Medical College, Vellore, India

^5^Duke University, Durham, NC, USA

^6^Fogarty International Center/National Institutes of Health, Bethesda, MD, USA

^7^Foundation for the NIH, Bethesda, MD, USA

^8^Haydom Lutheran Hospital, Haydom, Tanzania

^9^icddr, b, Dhaka, Bangladesh

^10^Institute of Medicine, Tribhuvan University, Kathmandu, Nepal

^11^Johns Hopkins University, Baltimore, MD, USA

^12^The Pennsylvania State University, University Park, PA, USA

^13^Temple University, Philadelphia, PA, USA

^14^Universidade Federal di Ceara, Fortaleza, Brazil

^15^University of Bergen, Norway

^16^University of Illinois at Chicago, IL, USA

^17^University of Venda, Thohoyandou, South Africa

^18^University of Virginia, Charlottesville, VA, USA

^19^Walter Reed/AFRIMS Research Unit, Kathmandu, Nepal

^20^Haukeland University Hospital, Bergen, Norway
